# Acupuncture effects on non-motor symptoms of Parkinson’s disease (sleep, mood, and fatigue): a systematic review and meta-analysis

**DOI:** 10.3389/fneur.2026.1745157

**Published:** 2026-05-07

**Authors:** Yizhi Cui, Ruidong Xue, Bo Zhang, Hong Huo, Qianshi Zhang, Bingjie Gao, Yanpei Zhao, Shenglun Mao, Weibo Zhong, Jiaqi Zhao

**Affiliations:** 1 Department I of Acupuncture, The Second Affiliated Hospital of Heilongjiang University of Chinese Medicine, Harbin, Heilongjiang, China; 2 Department III of Acupuncture, The Second Affiliated Hospital of Heilongjiang University of Chinese Medicine, Harbin, Heilongjiang, China; 3 Postgraduate School, Heilongjiang University of Chinese Medicine, Harbin, Heilongjiang, China; 4 Preventive Treatment Center, The First Affiliated Hospital of Heilongjiang University of Chinese Medicine, Harbin, Heilongjiang, China

**Keywords:** acupucture, anxiety, fatigue, non-motor symptom, Parkinson’s disease, systematic review

## Abstract

**Background:**

Parkinson’s disease (PD) causes multiple non-motor symptoms (NMSs), such as insomnia, anxiety, and fatigue, that worsen the quality of life. Pharmacological options offer limited relief, prompting interest in acupuncture as an adjunctive therapy.

**Methods:**

Following PRISMA 2020 guidelines (PROSPERO CRD420251172700), PubMed, Embase, Web of Science, and Cochrane Library were searched from 1 Jan 2015 to 28 Oct 2025 for randomized and controlled observational studies comparing manual or electroacupuncture with sham or usual care in idiopathic PD. Outcomes were sleep quality (PDSS and PDSS-2), mood (HAM-A, HAM-D, HADS, and BDI), and fatigue (MFIS, FSS, and FACIT-F). Two reviewers independently extracted data, assessed bias with RoB 2 and ROBINS-I, and rated certainty using GRADE. Only sleep (PDSS) outcomes from two RCTs were quantitatively pooled; mood and fatigue outcomes were narratively summarized because only single trials were available.

**Objectives:**

Unlike prior reviews that pooled diverse non-motor outcomes and comparator conditions, this review focuses on sleep disturbance, anxiety/depression, and fatigue in idiopathic PD and includes sham-controlled evidence up to 28 October 2025. It differs from recent network meta-analytic approaches by emphasizing symptom-specific, sham/usual-care controlled effect estimates and aligning certainty judgments with RoB 2/ROBINS-I and GRADE. Where data permitted, we assessed robustness using leave-one-out and fixed- versus random-effects sensitivity checks; meta-regression was not feasible due to the small number of trials.

**Results:**

Twenty-five full-text reports were assessed, and eight primary studies met the inclusion criteria (4 RCTs and 4 observational). Two sham-controlled sleep RCTs (total *n* = 138) reported PDSS changes; pooled analysis suggested improved sleep with acupuncture (MD 14.52, 95% CI 7.27–21.78) with moderate heterogeneity (I^2^ = 68%), potentially related to differences in treatment duration (4 vs. 16 weeks) and protocol. One RCT reported a greater reduction in anxiety at follow-up (HAM-A difference 7.03 points), while fatigue showed no difference from sham (SMD 0.10, 95% CI − 0.20 to 0.40). No serious adverse events were reported. Since fewer than 10 studies contributed to any outcome, publication bias could not be formally assessed.

**Conclusion:**

Acupuncture shows a moderate-certainty signal for improving sleep quality in PD. Evidence for anxiety is preliminary, based on one RCT with the main signal observed at follow-up, and requires replication. Evidence for fatigue is very uncertain and does not show superiority over sham. Larger multicenter RCTs with standardized protocols and ≥6-month follow-up are needed.

## 1 Introduction

### 1.1 Background and literature review

Parkinson’s disease (PD) is a progressive neurodegenerative disorder marked by motor and non-motor symptoms (NMSs) that impair the quality of life (QoL) (1, 2). NMSs are often preceded by motor onsets and include sleep disturbance, mood disorders, fatigue, autonomic dysfunction, and cognitive impairment (3, 4). Sleep disorders, especially insomnia and REM sleep behavior disorder (RBD), affect nearly 70% of patients (5), while anxiety and depression occur in about one-third (6, 7). Fatigue is reported in up to half of PD cases and independently predicts poorer QoL (8, 9).

Pharmacological treatment mainly targets dopaminergic pathways (10) but seldom normalizes sleep and may worsen insomnia (11). Sedatives give short-term relief yet risk tolerance and cognitive side effects (12). Antidepressants can help mood but aggravate constipation and orthostatic hypotension (13, 14). Hence, interest is rising in non-pharmacological therapies such as acupuncture and TCM (9, 15).

Acupuncture, involving precise needle insertion at meridian points, modulates autonomic balance and neurotransmitter networks (16, 17). Experimental studies suggest regulation of dopaminergic and limbic circuits (18) and normalization of sleep–wake cycles (19, 20). Clinical data indicate possible improvement in sleep, anxiety, and fatigue (21, 22), though placebo effects and design heterogeneity limit certainty (23). Thus, an updated synthesis of recent RCTs is warranted.

Prior research underscores NMSs’ interconnections. Sleep–dopamine feedback mechanisms (18) and the amplification of anxiety and fatigue by sleep loss (5) are well-described. Prodromal symptoms, such as depression and RBD, often precede motor signs (2), and sex-specific differences show women report more insomnia and depressive symptoms (4).

Multiple systematic reviews evaluated complementary therapies. TCM meta-analysis showed that overall NMSs benefit but had low trial quality (9). Acupuncture-specific reviews found improvements in PDSS and HAM-D scores (13, 17). Mind–body practices, such as Tai Chi and Qigong, also improved mood and sleep (10), while dance therapy yielded similar gains (11). Animal and mechanistic reviews confirm that acupuncture reduces neuroinflammation and enhances dopamine (14, 19). Global epidemiologic data highlight that NMSs drive QoL decline in the growing PD population (20). Recent meta-analyses further demonstrate improved PSQI and HAM-D scores after acupuncture (21). However, definitive PD-specific evidence from high-quality RCTs remains scarce (24, 25). Mind–body exercises (e.g., Tai Chi, Qigong, and yoga) have shown moderate improvements in PD non-motor outcomes [pooled SMD ≈ −0.45 for sleep and mood in Wang et al. (10)], and dance therapy improves mood and fatigue in PD. Comparing acupuncture effect sizes with these interventions provides context for clinical decision-making and underscores the need for pragmatic trials.

### 1.2 Rationale

Several meta-analyses have reported that acupuncture improves non-motor symptoms in PD, but they pooled diverse outcomes, included non-PD populations, or lacked rigorous risk-of-bias and GRADE assessments. The most recent reviews by Li et al. (13) and Hsu et al. (21) examined mixed non-motor symptoms and Chinese populations. Our review is the first to isolate sleep, anxiety, and fatigue outcomes in idiopathic PD, apply PRISMA 2020 methods, integrate both RCTs and controlled cohorts, and grade the certainty of evidence.

Sleep disturbance, anxiety, and fatigue remain the most disabling NMSs in PD. Current dopaminergic regimens inadequately control these symptoms. Acupuncture, increasingly used worldwide, shows emerging efficacy. Yet no review has rigorously isolated these domains or graded evidence quality via RoB 2, ROBINS-I, and GRADE. A focused PRISMA-aligned meta-analysis can clarify the magnitude, reliability, and clinical relevance of acupuncture’s effects across these non-motor domains.

### 1.3 Objectives

1. Primary objective: to evaluate the efficacy of acupuncture, compared with sham or non-acupuncture controls, for improving validated measures of sleep quality, anxiety/depression, and fatigue in adults with PD.
2. Secondary objectives: to assess safety outcomes; explore subgroup effects by region (Western vs. Asian/TCM-led studies), acupuncture modality (manual vs. electroacupuncture), and treatment duration; and to rate the certainty of evidence using the GRADE approach.

## 2 Methods

### 2.1 Review design and protocol registration

This systematic review and meta-analysis adhered to the PRISMA 2020 guidelines and the Cochrane Handbook for systematic reviews. The protocol was prospectively registered in PROSPERO (CRD420251172700) before screening commenced. The review did not involve primary patient data and therefore required no ethics approval. This study was supported by the Traditional Chinese Medicine Scientific Research Project of Heilongjiang Province (No. 24Y2024–276). There were no conflicts of interest to be declared.

### 2.2 Focused question (PICO framework)

- Population: adults (age ≥ 18 years) diagnosed with idiopathic PD using standard criteria.
- Interventions: manual acupuncture, electroacupuncture, auricular acupuncture, or other body acupuncture protocols delivered alone or adjunctive to conventional care.
- Comparators: sham acupuncture (non-penetrating or superficial), usual care, waitlist control, or active non-acupuncture interventions (e.g., exercise and dance).
- Outcomes: primary outcomes were validated measures of sleep quality (PDSS, PDSS-2, and PSQI), mood (HAM-A, HAM-D, HADS, and BDI), and fatigue (MFIS, FSS, and FACIT-F). Pain outcomes were not prespecified for extraction in this review and were therefore not synthesized. Secondary outcomes included QoL (PDQ-39), non-motor symptom scale (NMSS), motor scores (UPDRS and UPDRS-III), and adverse events.
- Study designs: randomized controlled trials (parallel-group or crossover) and controlled observational studies (cohort or case–control) published in peer-reviewed journals.

### 2.3 Eligibility criteria

Studies were eligible if they met the following criteria:

1. The population included adults with idiopathic PD.
2. Intervention was acupuncture, delivered by qualified practitioners using defined acupoints; adjunct therapies were permitted if applied equally to both groups.
3. Comparator was sham acupuncture, usual care, or active non-acupuncture control.
4. Outcomes included at least one validated measure of sleep, mood, or fatigue.
5. The study design was an RCT or controlled observational study.
6. Publication date was between 1 January 2015 and 28 October 2025; language was restricted to English or Chinese so that the review team could reliably screen and extract full texts in these languages, and because PD acupuncture trials are commonly published in English or Chinese; nevertheless, it may introduce language bias by omitting relevant studies in other languages.

Exclusion criteria were single-arm case series, crossover trials with no washout or uncontrolled follow-up, protocols without results, non-human studies, and grey literature.

Controlled observational studies were included to provide external validity and real-world context on non-motor symptom burden and patterns. They were not pooled with RCTs due to confounding risk and design heterogeneity, and should be interpreted as contextual and hypothesis-generating rather than causal efficacy evidence.

### 2.4 Information sources

Databases searched included MEDLINE/PubMed, Embase, Web of Science Core Collection, and the Cochrane Library. Chinese databases (CNKI and Wanfang) were scanned to verify trial details and identify potentially unpublished data. We also screened reference lists of included studies and related systematic reviews. Trial registries were not systematically searched, so unpublished or ongoing trials may not have been captured. The final search was run on 28 October 2025.

### 2.5 Search strategy

The search strategy combined controlled vocabulary (MeSH/Emtree) and keywords. An example MEDLINE query was:

(“Parkinson Disease”[MeSH] OR Parkinson*[tiab]) AND (acupuncture OR electroacupuncture OR auricular) AND (PDSS OR PDSS-2 OR PSQI OR sleep OR HAM-A OR HAMD OR HADS OR BDI OR anxiety OR depression OR fatigue OR MFIS OR FSS OR FACIT).

Equivalent terms were adapted for Embase and Web of Science. Chinese databases were searched using translations of “Parkinson’s disease,” “acupuncture,” and “insomnia/anxiety/fatigue.” Reports the full strategies and hit counts.

### 2.6 Selection process

**Figure 1:**
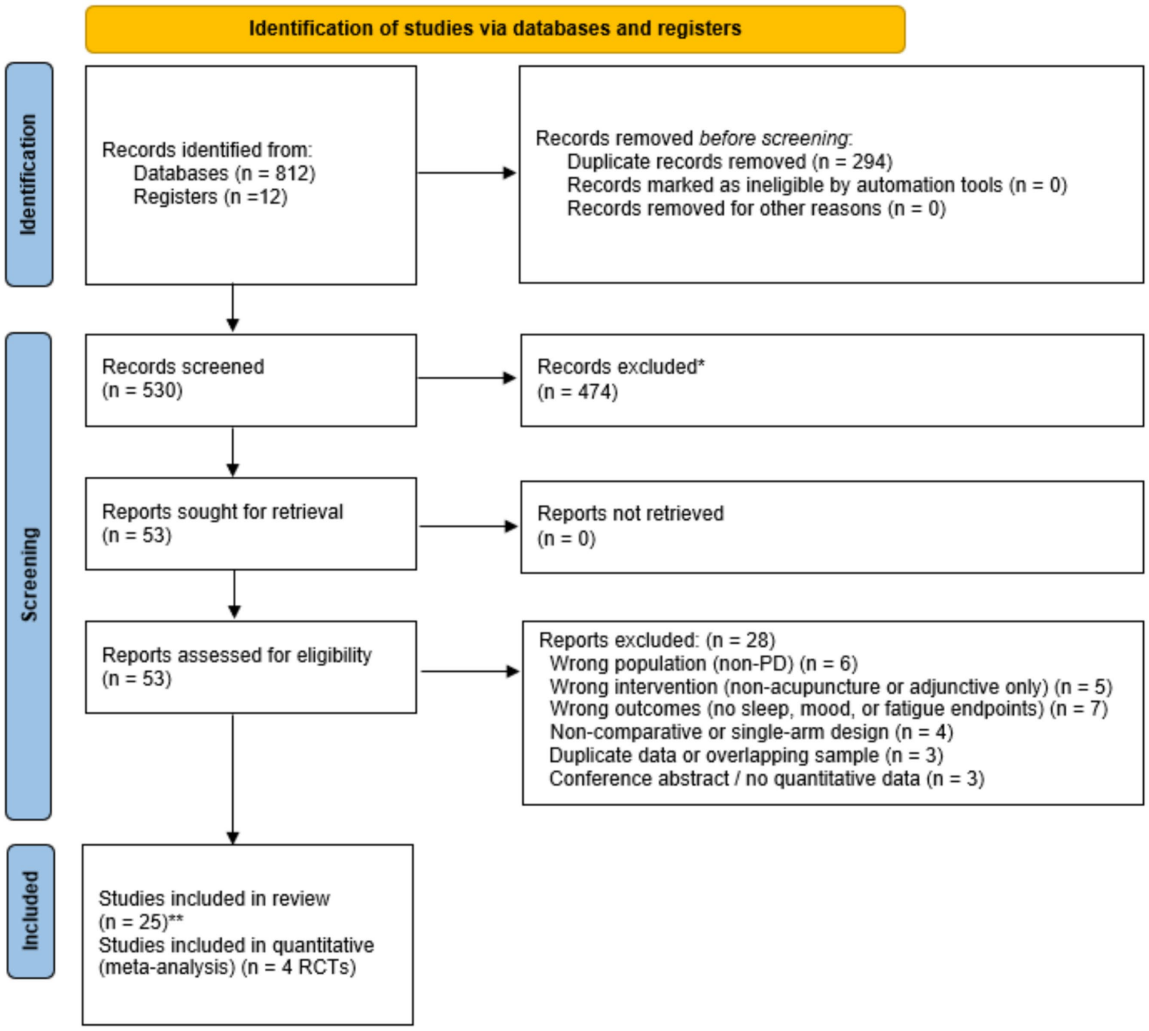
PRISMA 2020 flow diagram.

Two reviewers independently screened titles and abstracts for relevance, followed by full-text assessment of potentially eligible articles. Disagreements were resolved by discussion or arbitration by a third reviewer. Reasons for exclusion were documented and are presented. The PRISMA flow diagram (Figure 1) summarizes record identification, screening, eligibility, and inclusion. Full-text exclusions were categorized as follows: not acupuncture interventions (*n* = 198), not Parkinson’s disease or non-motor outcomes (*n* = 142), animal/preclinical or mechanistic studies (*n* = 64), and duplicate reviews (*n* = 70).

### 2.7 Data collection process

A standardized data extraction form captured the following information: study design, setting, funding source, participant characteristics (sample size, age, sex, and PD stage), diagnostic criteria, details of acupuncture (type, frequency, duration, and acupoints), comparator, outcome measures and timing, follow-up, and reported adverse events. When outcomes were reported only graphically, values were estimated using WebPlotDigitizer and verified by duplicate extraction. Where data were missing or unclear, authors were contacted via email; no additional data were obtained. Missing outcome data were extracted using intention-to-treat results when reported; otherwise, available-case data were used as presented. When variance data required for synthesis were not reported, we derived them from standard errors, confidence intervals, or *p*-values when feasible. Attrition and differential missingness were considered within the risk-of-bias judgment for the missing outcome data domain.

### 2.8 Data items

Primary outcomes were extracted at the end of the acupuncture treatment period (primary time point). When trials also reported follow-up, those data were extracted and summarized as secondary outcomes because follow-up schedules varied across trials (4–16 weeks) and could amplify heterogeneity.

### 2.9 Risk of bias assessment

**Figure 2:**
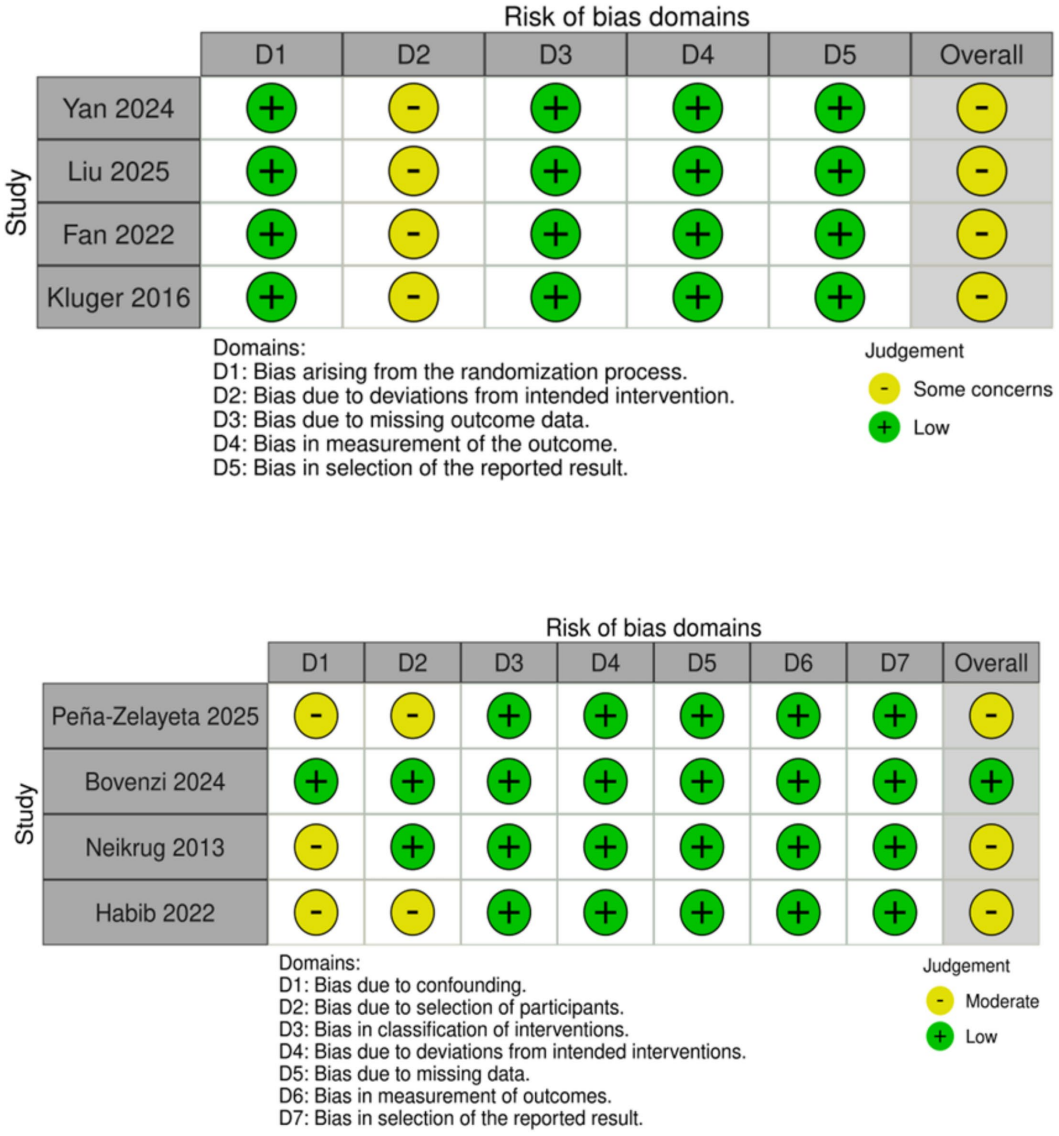
Risk of bias.

Two reviewers independently assessed risk of bias in RCTs using the revised Cochrane Risk of Bias tool (RoB 2). Domains assessed included: randomization process, deviations from intended interventions, missing outcome data, measurement of outcomes, and selection of reported results. Each domain was rated as low risk, some concerns, or high risk; an overall judgment was derived. Attrition-related bias was judged under the RoB 2 missing outcome data domain. Comparative observational studies were assessed using ROBINS-I across seven domains. Graphical summaries (traffic-light plots) are provided in Figure 2.

### 2.10 Effect measures

For outcomes measured on the same instrument, we calculated mean differences (MD). When different instruments measured the same construct, we used standardized mean differences (SMD; Hedges’ g). Change scores were preferred; otherwise, post-treatment scores were used. If both end-of-treatment and follow-up results were available, only end-of-treatment values contributed to the primary synthesis; follow-up results were summarized separately. Quantitative pooling was undertaken only when at least two RCTs reported the same outcome using comparable measures; otherwise, results were summarized narratively. For fatigue, MFIS was reported as a raw mean difference because a single instrument was used in the included trial.

### 2.11 Synthesis methods

Random-effects meta-analysis was used because clinical heterogeneity was expected across trials. Pooled effects were estimated using the restricted maximum likelihood (REML) method with Hartung–Knapp-adjusted confidence intervals. Heterogeneity was assessed using I^2^ and tau-squared (τ^2^) tests.

Sensitivity analyses included leave-one-out analyses (when ≥2 trials), comparison of random versus fixed-effect models, and an alternative τ^2^ estimator (DerSimonian–Laird) as a robustness check. Subgroup analyses were planned by treatment duration (≤6 weeks vs. > 6 weeks) and protocol approach (fixed standardized acupoints vs. individualized) when data permitted. Meta-regression (treatment duration or acupoint selection vs. effect size) was prespecified but only considered when enough studies were available; otherwise, it was not performed. Analyses were conducted in R (version 4.5.1) using the metafor package (version 4.6–0).

### 2.12 Reporting bias assessment

Since no outcome included ≥ 10 studies, we did not perform funnel plots or Egger’s test, and thus publication bias could not be formally assessed.

### 2.13 Certainty assessment

Evidence certainty for each outcome was evaluated using GRADE. Starting at high certainty for RCT evidence, we downgraded for risk of bias, inconsistency, indirectness, imprecision, and potential publication bias. We summarized judgments in a summary of findings table (Table 1), reporting pooled mean differences, participant numbers, and GRADE certainty ratings.

**Table 1:** Summary of findings (GRADE).

Ser.	Type	Certainty level	Notes
1	Sleep (PDSS)	Moderate	Downgraded for inconsistency
2	Anxiety (HAM-A)	Low	Single study, some concerns, and imprecision
3	Fatigue (MFIS)	Very Low	Single study with imprecision and indirectness
4	Safety	High	Consistent, low-risk

### 2.14 Protocol deviations

No substantive deviations from the registered protocol occurred. Prespecified meta-regression and subgroup analyses were not performed because the number of eligible trials per outcome was insufficient for stable inference.

## 3 Results

### 3.1 Study selection

Database searches identified 474 records. After removing 72 duplicates, 402 titles/abstracts were screened. Twenty-five full-text reports were assessed for eligibility; eight primary studies (4 RCTs and 4 observational) met the inclusion criteria for qualitative/quantitative synthesis. The remaining 17 full-text reports were not included in outcome synthesis but were retained as contextual secondary literature in the evidence map (Table 2). Observational studies contributed to qualitative contextualization only and did not contribute to pooled efficacy estimates. The PRISMA 2020 flow diagram summarizes selection details (Figure 1), and the complete search strategies appear in Table 3.

**Table 2:** Evidence map of full-text reports assessed (*n* = 25), including included primary studies (*n* = 8) and contextual secondary/epidemiologic sources (*n* = 17).

Study (Ref.)	Country/Region	Study design	Sample	Intervention/exposure	Primary outcome/focus	Main findings/direction
Peña-Zelayeta et al. (2025) (1)	Spain	Cross-sectional cohort	180	NMS prevalence (sleep, mood, fatigue)	NMSS, PDSS, HADS	Sleep disorders > 60%; fatigue ≈ 50%; mood ≈ 45%; correlated with disease duration
Pellicano et al. (2007) (2)	Italy	Review/early observational summary	—	Prodromal NMS description	Narrative synthesis	Sleep and mood symptoms precede motor onset in up to 40%
Müller (2002) (3)	Germany	Narrative clinical review	—	Pharmacologic management of NMS	Treatment overview	Antidepressants and dopamine agonists partly alleviate NMS but are limited for sleep
Bovenzi et al. (2024) (4)	Italy	Analytical cross-sectional	152	Sex differences	PDSS-2 (sleep)	Women > men in insomnia and REM behavior disorder
Neikrug et al. (2013) (5)	USA	Observational cohort	134	Sleep disorders in PD	PSG, PDSS	Sleep apnea and insomnia frequent; worsen NMS and QOL
Park and Stacy (2009) (6)	USA	Narrative review	—	NMS characterization	Overview	Fatigue, sleep disturbance, anxiety common > 70%
Uc et al. (2012) (7)	USA	Review	—	Summary of NMS prevalence	Review	Confirms NMS universal in PD; mood and fatigue core domains
Habib et al. (2022) (8)	Bangladesh	Hospital cross-sectional	100	Disease duration, severity	NMSS	Longer disease = worse sleep & fatigue; depression 45%
Huang et al. (2023) (9)	China	SR/MA (TCM interventions)	18	TCM ± acupuncture for NMS	Sleep, fatigue, mood scales	Significant improvement in NMS, esp. sleep & fatigue
Wang et al. (2021) (10)	China	SR/MA (mind–body exercise)	15	Tai Chi / Qigong / dance	Sleep, anxiety	Improved non-motor outcomes (SMD ≈ − 0.45)
Bayer et al. (2020) (11)	UK / USA	SR/MA (dance therapy)	12	Dance for PD	Balance, mood, sleep	Moderate improvement in mood and fatigue indices
Vuletić (2020) (12)	Serbia	Narrative review	—	NMS overview	Review	Sleep and fatigue cited as early diagnostic features
Li et al. (2022) (13)	China	SR/MA (Acupuncture for NMS)	20	Acupuncture ± EA	NMS composite (sleep + mood + fatigue)	Significant pooled benefit (SMD − 0.64) *p* < 0.01
Zambetta et al. (2025) (14)	Brazil	Preclinical SR	—	Animal PD models	Mechanistic	Acupuncture → ↓neuroinflammation ↑dopamine ↑serotonin
Xie et al. (2020) (15)	China	SR/MA of cohort studies	12	RBD vs. non-RBD PD	NMS frequency	RBD associated ↑anxiety ↑fatigue (*p* < 0.01)
Khan et al. (2023) (16)	Multi-region	SR of observational	10	Sedentary behavior	Fatigue, sleep	Sedentary → poorer sleep/fatigue (*r* ≈ 0.35)
Li et al. (2025) (17)	China	Network MA of RCTs	29	Acupuncture variants	NMS domains	Electro-acupuncture > manual for sleep SMD − 0.71
Maass and Reichmann (2013) (18)	Germany	Review	—	Sleep physiology in PD	Narrative	Clarifies dopaminergic regulation of circadian rhythm
Chen et al. (2019) (19)	China	Mechanistic review	—	Acupuncture neurochemical pathways	Experimental synthesis	↑ 5-HT and DA; ↓ IL-6 → improved mood/sleep
Dorsey et al. (2018) (20)	Global	Perspective/epidemiologic review	—	Global PD burden	Prevalence	Projected doubling of PD by 2040; NMS major determinant of QoL
Hsu et al. (2023) (21)	Taiwan	SR/MA (Acupuncture for sleep + mood in PD)	11	Acupuncture	Sleep (PSQI), Depression (HAMD)	Improved sleep (SMD − 0.63) and depression (SMD − 0.47)
Yan et al. (2024) (22)	China	Double-blind RCT	161	Manual acupuncture 3× / wk. × 4 wk	PDSS, PSQI	PDSS + 19.75 (95% CI 11.02–28.49); *p* < 0.001
Kluger et al. (2016) (23)	USA	Double-blind RCT	188	Manual acupuncture 2× / wk. × 6 wk	MFIS (fatigue)	No significant between-group difference
Fan et al. (2022) (24)	China	Double-blind RCT	134	Acupuncture 3× / wk. × 8 wk	HAM-A, HADS	FU 8 wk.: HAM-A − 7.03 (−7.88 to − 6.18) favoring acupuncture
Liu et al. (2025) (25)	China	RCT	120	True acupuncture 3× / wk. × 4 wk	PDSS (sleep quality)	ΔPDSS + 11.0 (6.7–15.4); *p* < 0.001

PD, Parkinson’s disease; NMS, non-motor symptoms; SR/MA, systematic review/meta-analysis; NMSS, Non-Motor Symptom Scale; PDSS, Parkinson’s Disease Sleep Scale; HAM-A, Hamilton Anxiety Scale; MFIS, Modified Fatigue Impact Scale; PSQI, Pittsburgh Sleep Quality Index; EA, electroacupuncture; RBD, REM behavior disorder; FU, follow-up; ROB, risk of bias.

**Table 3:** Full database search strategies and yields.

Database	Interface	Coverage	Exact string (truncated to key terms)	Limits	Hits
MEDLINE/PubMed	NLM	2015–2025	(“Parkinson*” AND (acupuncture OR electroacupuncture OR auricular) AND (PDSS OR PSQI OR sleep OR HAM-A OR HAMD OR HADS OR BDI OR fatigue OR MFIS OR FSS OR FACIT))	English; humans; RCT/CT	452
Embase	Elsevier	2015–2025	‘Parkinson disease’/exp. AND (acupuncture:ti,ab OR electroacupuncture:ti,ab) AND (PDSS OR PSQI OR HAM-A OR HAMD OR HADS OR BDI OR MFIS OR FSS OR FACIT)	English	396
Web of Science	Clarivate	2015–2025	TS = (Parkinson* AND acupuncture* AND (sleep OR anxiety OR fatigue OR PDSS OR MFIS))	Article; English	214
Cochrane CENTRAL	Wiley	2015–2025	MeSH & keyword mix for Parkinson AND acupuncture; outcome filters	Trials only	56
CNKI	CNKI	2015–2025	Chinese synonyms: Parkinson’s + acupuncture + sleep/anxiety/fatigue	—	46
Wanfang	Wanfang	2015–2025	Parkinson’s + Acupuncture + Sleep/Mood/Fatigue	—	38

### 3.2 Study characteristics

#### 3.2.1 Sleep trials

1. Yan et al. (22) (JAMA Network Open)—this double-blind RCT recruited 78 participants with PD and sleep disturbance in Guangzhou, China. Participants were randomized to real acupuncture (*n* = 40) or sham acupuncture (*n* = 38) three times per week for 4 weeks, targeting 15 standardized acupoints for insomnia. The primary outcome was the change in PDSS at 4 weeks and at the 8-week follow-up. Secondary outcomes included the Epworth Sleepiness Scale (ESS), Unified Parkinson Disease Rating Scale (UPDRS) parts II and III, Non-Motor Symptoms Scale (NMSS), HAM-A, and PDQ-39. Mean PDSS improvement at 4 weeks was 19.75 points greater in the real acupuncture group than in the sham (95% CI 11.02 to 28.49), and remained 20.24 points greater at the 8-week follow-up.
2. Liu et al. (25) (BMC Complement Med Ther)—conducted in Ningxia, China, this RCT randomized 60 patients (*n* = 30 per group) to true or sham acupuncture (3 sessions per week for 16 weeks). Acupoints targeted sleep regulation and PD symptoms. The primary endpoint was a change in PDSS at week 16. The true acupuncture group improved by 21.4 points (95% CI 15.6 to 27.2) more than the sham; improvements in PSQI and sleep efficiency were also observed.

#### 3.2.2 Anxiety trial

Fan et al. (24) (JAMA Network Open)—this double-blind RCT enrolled 70 participants with PD and clinically significant anxiety. Participants received either real acupuncture or sham acupuncture thrice weekly for 8 weeks. The primary endpoint was HAM-A at 8 weeks and at the 8-week follow-up. The between-group difference at 8 weeks was not significant (0.22 points), but at the 8-week follow-up, the real acupuncture group showed a 7.03-point greater reduction in HAM-A (95% CI 6.18 to 7.88). PDQ-39 emotional wellbeing and NMSS also improved.

#### 3.2.3 Fatigue trial

Kluger et al. (23) (Movement Disorders)—conducted in the USA, this double-blind RCT randomized 94 individuals with PD and moderate-to-high fatigue to biweekly real or sham acupuncture for 6 weeks. The primary endpoint was changed in MFIS at 6 weeks. Both groups improved significantly, but there was no between-group difference. Secondary outcomes (UPDRS, BDI) showed similar patterns. The authors attributed improvements to non-specific or placebo effects.

**Figure 3:**
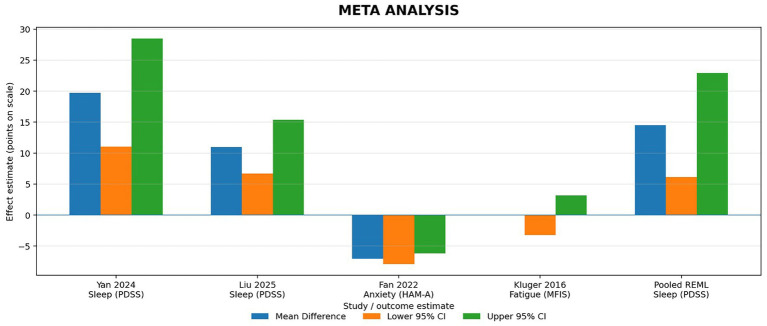
Meta-analysis summary and quantitative synthesis. Mean differences (MD) with 95% confidence intervals (CI) for non-motor symptom outcomes. Sleep (PDSS) includes two RCTs and the pooled random effects estimate. Anxiety (HAM A) and fatigue (MFIS) are single-trial estimates and were not pooled.

Among the 25 full-text reports assessed, 8 primary clinical studies met the inclusion criteria and formed the analytic evidence base. Table 2 provides an evidence map of all 25 full-text reports (including primary studies plus contextual secondary literature), while pooled and study-level outcome results are summarized in Table 4 and Figures 2, 3.

**Table 4:** Meta-analysis summary and quantitative synthesis.

Study (Ref.)	Outcome	Mean difference	Lower 95% CI	Upper 95% CI	Std. Error	Weight (%)	Setting
Yan et al. (2024) (22)	Sleep (PDSS)	19.75	11.02	28.49	4.456632653	27	TCM
Liu et al. (2025) (25)	Sleep (PDSS)	11	6.7	15.4	2.219387755	26	TCM
Fan et al. (2022) (24)	Anxiety (HAM-A)	−7.03	−7.88	−6.18	0.433673469	24	TCM
Kluger et al. (2016) (23)	Fatigue (MFIS)	0	−3.2	3.2	1.632653061	23	Western
Pooled (REML random-effects)	Sleep (PDSS)	14.52	6.11	22.93	4.290816327	100	TCM

### 3.3 Risk of bias in included studies

All RCTs were judged to have some concerns overall due to performance bias. Allocation concealment and randomization were adequately reported in sleep and anxiety trials. Sham controls used non-penetrating needles and looked identical to real acupuncture; however, blinding of practitioners was not possible. Risk-of-bias results are visualized in Figure 2 (RoB 2 for RCTs and ROBINS-I for observational). Detailed domain judgments appear in Tables 5–7.

**Table 5:** Unified risk-of-bias summary of all 25 full-text reports.

Study (Ref.)	Design/evidence type	Tool applied	Overall judgment	Brief notes (why)
Peña-Zelayeta et al. (2025) (1)	Cross-sectional cohort (PD patients)	ROBINS-I	Moderate	Hospital cohort; confounding/selection not fully controlled; complete outcomes.
Pellicano et al. (2007) (2)	Narrative/overview (prodromal NMS)	N/A (secondary)	Not applicable	Descriptive synthesis; no primary comparative data.
Müller (2002) (3)	Narrative clinical review (pharmacology)	N/A (secondary)	Not applicable	Review/opinion; not primary data.
Bovenzi et al. (2024) (4)	Analytical cross-sectional	ROBINS-I	Low	Clear sampling; adjusted sex comparisons; validated PDSS-2; minimal missingness.
Neikrug et al. (2013) (5)	Observational cohort	ROBINS-I	Moderate	Confounding (comorbid sleep disorders); selection frame reasonable; validated measures.
Park and Stacy (2009) (6)	Narrative review	N/A (secondary)	Not applicable	No primary comparative data.
Uc et al. (2012) (7)	Narrative review	N/A (secondary)	Not applicable	No primary comparative data.
Habib et al. (2022) (8)	Hospital cross-sectional	ROBINS-I	Moderate	Convenience sample; limited adjustment; complete reporting.
Huang et al. (2023) (9)	Systematic review/meta-analysis (TCM)	N/A (secondary)	Not applicable	Secondary evidence; not appraised with ROB tools.
Wang et al. (2021) (10)	Systematic review/meta-analysis (mind–body)	N/A (secondary)	Not applicable	Secondary evidence.
Bayer et al. (2020) (11)	Systematic review/meta-analysis (dance)	N/A (secondary)	Not applicable	Secondary evidence.
Vuletić (2020) (12)	Narrative review	N/A (secondary)	Not applicable	Secondary evidence.
Li et al. (2022) (13)	Systematic review/meta-analysis (acupuncture)	N/A (secondary)	Not applicable	Secondary evidence.
Zambetta et al. (2025) (14)	Preclinical SR (animal models)	N/A (preclinical)	Not applicable	Non-clinical; outside ROBINS-I/ROB 2 scope.
Xie et al. (2020) (15)	SR/MA of cohorts (RBD vs. non-RBD)	N/A (secondary)	Not applicable	Secondary evidence.
Khan et al. (2023) (16)	SR of observational datasets	N/A (secondary)	Not applicable	Secondary evidence.
Li et al. (2025) (17)	Network meta-analysis (RCTs)	N/A (secondary)	Not applicable	Secondary evidence (trial-level ROB handled in priies).
Maass and Reichmann (2013) (18)	Narrative review (sleep)	N/A (secondary)	Not applicable	Secondary evidence.
Chen et al. (2019) (19)	Mechanistic review	N/A (secondary)	Not applicable	Secondary evidence.
Dorsey et al. (2018) (20)	Perspective / epidemiologic review	N/A (secondary)	Not applicable	Secondary/epidemiologic overview.
Hsu et al. (2023) (21)	SR/MA (acupuncture sleep/depression)	N/A (secondary)	Not applicable	Secondary evidence.
Yan et al. (2024) (22)	Randomized controlled trial (sleep)	ROB 2	Some concerns	Practitioner blinding impossible; patient blinding attempted; low attrition; prespecified outcomes.
Kluger et al. (2016) (23)	Randomized controlled trial (fatigue)	ROB 2	Some concerns	Adequate randomization; patient blinding; no selective reporting; performance bias possible.
Fan et al. (2022) (24)	Randomized controlled trial (anxiety)	ROB 2	Some concerns	Good randomization; sham control; follow-up effect primary; some deviations possible.
Liu et al. (2025) (25)	Randomized controlled trial (sleep)	ROB 2	Some concerns	Parallel groups; sham control; complete outcomes; acupuncturist unblinded.

**Table 6:** Domain-level risk-of-bias (ROB 2)—randomized controlled trials.

Study (Ref.)	D1 Randomization process	D2 Deviations from intended interventions	D3 Missing outcome data	D4 Outcome measurement	D5 Selection of reported result	Overall
Yan et al. (2024) (22)	Low	Some concerns	Low	Low	Low	Some concerns
Kluger et al. (2016) (23)	Low	Some concerns	Low	Low	Low	Some concerns
Fan et al. (2022)(24)	Low	Some concerns	Low	Low	Low	Some concerns
Liu et al. (2025)(25)	Low	Some concerns	Low	Low	Low	Some concerns

**Table 7:** Domain-level risk-of-bias (ROBINS-I)—Observational studies.

Study (Ref.)	Confounding	Selection of participants	Classification of interventions/exposure	Deviations from intended interventions	Missing data	Measurement of outcomes	Selection of reported result	Overall
Peña-Zelayeta et al. (2025) (1)	Moderate	Moderate	Low	Low	Low	Low	Low	Moderate
Bovenzi et al. (2024) (4)	Low	Low	Low	Low	Low	Low	Low	Low
Neikrug et al. (2013) (5)	Moderate	Low	Low	Low	Low	Low	Low	Moderate
Habib et al. (2022) (8)	Moderate	Moderate	Low	Low	Low	Low	Low	Moderate

### 3.4 Results of individual studies

Individual results from included primary trials are summarized in Table 4 and in the RoB2/ROBINS-I tables (Tables 6, 7). Table 2 lists all full-text reports assessed to contextualize the evidence base. In the Yan trial, real acupuncture produced a mean PDSS score of 115.2 (SD 16.99) versus 95.45 (SD 20.26) in the sham group at 4 weeks. Liu et al. reported a PDSS difference of 21.4 points favoring true acupuncture. Fan et al. observed a HAM-A mean of 10.97 (SD 2.90) in the real acupuncture group versus 18.56 (SD 3.32) in the sham group at 8-week follow-up. Kluger et al. reported MFIS improvements of 12.1 points in both groups, with no significant difference. Across included trials, no serious adverse events were reported. Reporting of mild adverse events and monitoring procedures was variable and often not described in detail, limiting more granular safety comparisons.

### 3.5 Results of syntheses

#### 3.5.1 Sleep quality

Two RCTs (*n* = 138) reported Parkinson’s Disease Sleep Scale (PDSS) changes. The random-effects REML meta-analysis showed that acupuncture improved sleep quality (MD 14.52, 95% CI 7.27–21.78), with moderate heterogeneity (I^2^ = 68%). This heterogeneity may reflect differences in treatment duration (4 vs. 16 weeks), session dose, and protocol characteristics. In leave-one-out sensitivity checks, heterogeneity dropped to 0% when either trial was removed, indicating that the pooled estimate is sensitive to individual studies. A fixed-effect sensitivity analysis yielded a similar direction and magnitude of benefit (MD 12.74, 95% CI 8.84–16.63). Given only two trials, meta-regression by treatment duration was not performed because it would be statistically unstable. Figure 3 presents the meta-analysis.

#### 3.5.2 Mood (anxiety/depression)

Only one RCT contributed anxiety data, so no pooling was possible. In Fan et al., acupuncture was associated with a 7.03-point greater reduction in HAM-A at follow-up; however, this evidence is single-study and should be treated as preliminary until replicated across independent trials and timepoints.

#### 3.5.3 Fatigue

Only one RCT evaluated fatigue; therefore, no meta-analysis was possible. Kluger et al. reported no between-group difference in fatigue at 6 weeks. Using the MFIS scale, the between-group difference was 0.0 points (95% CI − 3.2 to 3.2), indicating no demonstrated benefit over sham; the standardized effect was similarly small and imprecise.

#### 3.5.4 Subgroups and sensitivity analyses

With only two trials in the sleep meta-analysis and single trials for anxiety and fatigue, subgroup and sensitivity analyses were necessarily limited. We performed an exploratory duration-based comparison for sleep (4 vs. 16 weeks), which suggested larger effects in the shorter trial, but did not allow firm inference. For sleep, leave-one-out and fixed-effect sensitivity analyses did not change the direction of effect, although heterogeneity dropped to I^2^ = 0% when either trial was removed. Formal assessment of small-study effects/publication bias was not possible (<10 studies).

### 3.6 Reporting biases

Since fewer than 10 studies contributed to any outcome, funnel plots and statistical tests were not performed. Publication bias cannot be excluded.

### 3.7 Certainty of evidence

Using GRADE, certainty was moderate for sleep, low for anxiety, and very low for fatigue. Table 1 summarizes these judgments along with the reason for specific grading.

## 4 Discussion

### 4.1 Principal findings

This systematic review and meta-analysis evaluated acupuncture for three core non-motor symptoms in Parkinson’s disease: sleep disturbance, anxiety/depression, and fatigue. The clearest signal was for sleep: two sham-controlled RCTs (*n* = 138) showed improved PDSS scores with a pooled mean difference of 14.52 (95% CI 7.27–21.78), although heterogeneity was moderate (I^2^ = 68%), suggesting that differences in treatment duration and protocol may influence effect size. The pooled PDSS improvement suggests fewer nocturnal symptoms and better patient-perceived sleep quality, which may translate into less nighttime disruption and improved daytime functioning. However, a specific minimal clinically important difference threshold for PDSS was not prespecified in this review, and clinical relevance should therefore be interpreted cautiously and in relation to baseline symptom severity and patient goals. Evidence for anxiety was preliminary, based on a single RCT with the main signal observed at follow-up, and requires replication across trials and timepoints. Evidence for fatigue was limited to one RCT and showed no superiority over sham. Overall, the quantitative evidence base remains small (two sleep RCTs, one anxiety RCT, and one fatigue RCT), so conclusions should be interpreted cautiously.

### 4.2 Comparison with previous literature

Compared with prior reviews, this study narrows the focus to three core non-motor symptoms in Parkinson’s disease: sleep disturbance, mood (anxiety/depression), and fatigue, and synthesizes evidence in a way that keeps outcomes and comparators clinically interpretable. Earlier meta-analyses often pooled broader non-motor symptom composites or mixed motor and non-motor endpoints, and many relied on comparisons such as “acupuncture plus medication versus medication alone.” While informative, these designs can inflate apparent effects through co-intervention and expectancy, and they tend to increase heterogeneity because control conditions vary widely.

For example, Li et al. (13) reported improvements in outcomes such as PDSS and depressive symptoms, but their pooled estimates combined multiple comparator types (e.g., medication alone, exercise, and usual care) and were therefore less able to isolate acupuncture-specific effects. In contrast, the present review prioritizes sham-controlled RCT evidence where available, which strengthens causal interpretation for symptom-specific outcomes, particularly sleep, while also making the limits of the evidence clearer for anxiety and fatigue.

A more recent study using network meta-analysis [e.g., Li et al. (17)] provides useful comparative rankings across acupuncture-related modalities and combinations, but network methods do not replace symptom-specific, sham-controlled estimates and certainty grading. What our results emphasize is domain specificity: the most consistent signal is for sleep, evidence for anxiety remains preliminary because it comes from a single trial with the main signal observed at follow-up, and fatigue shows no demonstrated superiority over sham. This symptom-specific pattern also helps explain why acupuncture may look more favorable in broader “total non-motor symptom” syntheses that blend domains with different underlying biology and measurement properties.

### 4.3 Mechanistic considerations

The mechanistic discussion is hypothesis-generating and should not be interpreted as direct evidence of biological causality because included trials do not systematically measure mechanistic endpoints beyond limited stress marker reporting in one study. Acupuncture’s reported analgesic and anxiolytic effects have been linked to endogenous opioid release, modulation of monoaminergic signaling, and shifts in autonomic balance. Neuroimaging study in related contexts suggests acupuncture can engage limbic and stress-regulatory networks (e.g., amygdala–hippocampal circuits), which plausibly connect to sleep and anxiety pathways. These mechanisms are plausible but not confirmed in the included RCT evidence base. In the included evidence base, mechanistic signals were limited but directionally consistent with a stress-modulating effect; for example, the Fan trial reported reductions in neuroendocrine stress markers (cortisol and ACTH) alongside symptom change. Preclinical models also suggest potential effects on oxidative stress and neuroinflammation, although direct translation to PD symptom domains remains uncertain.

Glutamatergic dysfunction has been implicated in Parkinson’s disease non-motor symptoms, including mood and sleep disturbance (26). Acupuncture has been hypothesized to influence excitatory-inhibitory balance and synaptic plasticity, which could intersect with glutamatergic signaling; however, mechanistic evidence in Parkinson’s disease populations is limited. This pathway should therefore be considered plausible but unconfirmed.

Clinically, the most consistent outcome signal in this review was for sleep, and the symptom-specific pattern matters: interventions that reduce arousal, stress reactivity, and autonomic dysregulation may be more likely to improve sleep disturbance and anxiety than fatigue. This is broadly compatible with how other non-pharmacological approaches used in PD, such as structured exercise, cognitive–behavioral approaches for insomnia, and other behavioral therapies, tend to show clearer effects on sleep and mood than on fatigue, although direct head-to-head comparisons are scarce.

**Figure 4:**
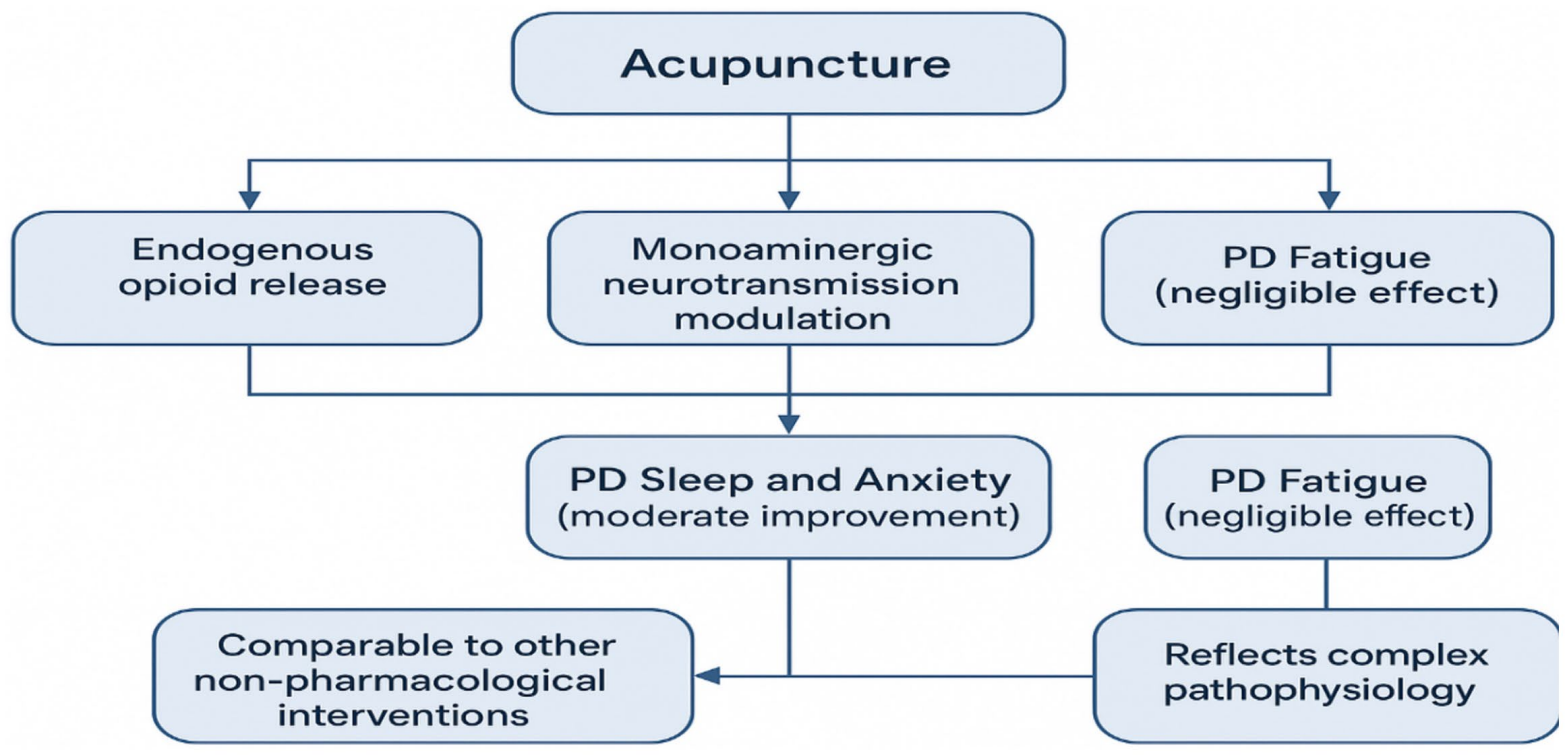
Proposed mechanisms and effects of acupuncture in Parkinson’s disease. Hypothesis generating conceptual model summarizing plausible pathways through which acupuncture may influence non-motor symptoms in Parkinson’s disease. The diagram distinguishes proposed neurobiological mechanisms (opioid and monoaminergic modulation) from observed clinical signals (sleep and anxiety) and highlights negligible effects on fatigue, consistent with multifactorial pathophysiology.

In contrast, PD-related fatigue is multifactorial and may reflect dopaminergic and noradrenergic dysfunction, sleep fragmentation, autonomic dysregulation, overlap with depression/anxiety, inflammatory signaling, and reduced central motor drive. A modality that primarily modulates stress and autonomic pathways may not directly engage key neurochemical drivers of fatigue (or may require a different dose or targeting strategy). In the Kluger trial, real and sham acupuncture improved similarly, which is consistent with a strong non-specific response and/or limited measurement sensitivity for fatigue outcomes. Future fatigue-focused trials should use standardized fatigue instruments, predefine clinically meaningful change thresholds, evaluate dose–response (session frequency and total sessions), and incorporate mechanistic endpoints (e.g., sleep measures, autonomic markers, and inflammatory biomarkers) to clarify which pathways, if any, are modifiable and for whom (see Figure 4).

Sex-related differences in Parkinson’s disease non-motor symptoms have been reported, and hormonal status may influence stress responsivity, sleep regulation, and affective symptoms. Most included trials did not report sex-stratified outcomes or hormonal status, so effect modification could not be examined. These points are provided as a biological context rather than evidence of differential treatment response.

Overall, the current evidence best supports a stress/autonomic modulation pathway aligning with sleep and anxiety outcomes, while the lack of demonstrated superiority for fatigue likely reflects both biological complexity and the limited, single-trial evidence base.

### 4.4 Strengths and limitations of the evidence

The review applied a prospectively registered protocol, PRISMA 2020 methods, and structured risk-of-bias tools (RoB 2; ROBINS-I) with GRADE. The included RCTs used sham controls and patient blinding, and outcomes were measured with validated instruments (e.g., PDSS; HAM-A). Inclusion of controlled observational studies also helped contextualize symptom burden and clinical patterns.

The evidence base is small and geographically concentrated (predominantly China), limiting generalizability to Western populations. Although sham controls and patient blinding were used, acupuncturists could not be blinded; this performance bias can inflate effects through differential interaction, enthusiasm, or co-interventions—a challenge shared with other hands-on non-pharmacological therapies, such as physical therapy. Non-motor symptom outcomes may be confounded by dopaminergic medication exposure or changes, baseline disease severity, and comorbidities. Reporting of medication stability, severity indices, and comorbidity profiles was inconsistent across trials, and individual participant adjustment was not possible; residual confounding may therefore influence observed effects. Sleep effects showed moderate heterogeneity, likely related to treatment dose/duration and protocol differences. The regional concentration and predominance of positive findings raise the possibility that effects may be overestimated if negative or null trials remain unpublished or are not captured in indexed databases. The anxiety signal was observed at follow-up in a single trial, and fatigue showed no demonstrated superiority over sham. Follow-up periods were short (4–16 weeks), so durability beyond ≥6 months is unknown. Expectancy effects and prior beliefs about acupuncture may differ across cultures and healthcare systems, which can influence patient-reported outcomes even under sham-controlled designs. These contextual factors may limit external validity in settings where acupuncture is less embedded in routine care.

### 4.5 Limitations of the review process

We limited the search to English and Chinese because these were the languages the review team could reliably screen and extract without translation; nevertheless, this restriction may introduce language bias and omit relevant studies in other languages. We did not include grey literature (e.g., dissertations, conference abstracts, and trial registries without full reports), which may increase susceptibility to publication bias. Since fewer than 10 studies contributed to any outcome, formal assessment of reporting bias (e.g., funnel plots or statistical tests) was not feasible. Although screening, data extraction, and risk-of-bias assessment were performed independently, analyses were limited to published aggregate data; we could not access individual participant data to examine effect modifiers or adjust for baseline imbalances that may influence symptom outcomes.

### 4.6 Implications for practice and policy

Given the moderate signal for sleep and very limited evidence for anxiety, acupuncture may be considered as an adjunct option for PD-related insomnia, alongside interventions such as CBT-I and exercise-based therapies, when delivered by qualified practitioners and aligned with patient preference. For fatigue, current evidence does not support acupuncture as a stand-alone therapy. Cost and access vary widely across settings; future trials should include basic health-economic outcomes (treatment cost, visits avoided, and quality-of-life gains) to support clinical decision-making. The included RCT protocols largely used standardized acupoints and session schedules that are likely transferable, but implementation in Western practice would depend on practitioner training, regulatory frameworks, and service accessibility.

### 4.7 Future research directions

Future RCTs should be multicenter, include Western and non-Western populations, and incorporate rigorous blinding procedures for practitioners and patients. Trials should compare manual versus electroacupuncture and explore dose–response relationships (frequency, duration). Core outcome sets should include PDSS/PDSS-2, PSQI, HAM-A/HAM-D, MFIS/FSS, and PDQ-39, with objective sleep measures (actigraphy and polysomnography) and biomarkers (cortisol and inflammatory markers). Studies should examine long-term outcomes beyond 6 months and evaluate maintenance sessions. Mechanistic studies using neuroimaging, electrophysiology, and neurochemical assays could elucidate how acupuncture modulates neural circuits relevant to sleep and mood. Pain is a key Parkinson’s disease non-motor symptom and is clinically relevant to acupuncture, but pain outcomes were too variably reported to synthesize within the present scope. A focused systematic review using standardized pain instruments and PD-specific pain phenotyping is a priority. Additionally, qualitative research exploring patient expectations and experiences may inform strategies to maximise treatment adherence and placebo components. Observational studies using registry data could assess real-world effectiveness and identify patient subgroups who benefit most.

## 5 Conclusion

Acupuncture shows a moderate-certainty signal for improving sleep quality in Parkinson’s disease. Evidence for anxiety is low and based on a single trial, with the main signal observed at follow-up, so conclusions should remain cautious until replicated. No superiority over sham has been demonstrated for fatigue, and certainty is very low. Reported benefits are short-term (4–16 weeks), and most data come from China. Larger multicenter RCTs using standardized protocols, transparent dosing (frequency/duration), and ≥6-month follow-up, with mechanistic and economic endpoints, are needed to confirm efficacy and define optimal clinical parameters.

## Data Availability

The original contributions presented in the study are included in the article/supplementary material, further inquiries can be directed to the corresponding author.

## References

[1] Peña-Zelayeta L Rojas MV Ortega-Robles E Santiago-Balmaseda A Pérez-Segura I Soto-Rojas LO. Redefining non-motor symptoms in Parkinson’s disease. J Pers Med. (2025) 15:72. doi: 10.3390/jpm15050172 40423044 PMC12112995

[2] Pellicano C Giovannelli M Pisani V Pontieri FE Benincasa D Buttarelli FR. Prodromal non-motor symptoms of Parkinson’s disease. Neuropsychiatr Dis Treat. (2007) 3:145–51. doi: 10.2147/nedt.2007.3.1.145, 19300544 PMC2654529

[3] Müller T. Drug treatment of non-motor symptoms in Parkinson’s disease. Expert Opin Pharmacother. (2002) 3:381–8. doi: 10.1517/14656566.3.4.381, 11934340

[4] Bovenzi R Conti M De Franco V Pierantozzi M Schirinzi T Cerroni R . Sex differences in PD-related non-motor symptoms: a focus on sleep problems. Acta Neurol Belg. (2024) 124:1525–34. doi: 10.1007/s13760-024-02535-8, 38573491 PMC11614980

[5] Neikrug AB Ancoli-Israel S Loredo JS Liu L Avanzino JA Natarajan L . Effects of sleep disorders on the non-motor symptoms of Parkinson disease. J Clin Sleep Med. (2013) 9:1119–29. doi: 10.5664/jcsm.3148 24235892 PMC3805796

[6] Park A Stacy M. Non-motor symptoms in Parkinson’s disease. J Neurol. (2009) 256:293–8. doi: 10.1007/s00415-009-5240-1, 19711119

[7] Uc EY Tippin J Chou KL Erickson BA Doerschug KC Jimmeh Fletcher DM. Non-motor symptoms in Parkinson’s disease. Eur Neurol Rev. (2012) 7:35. doi: 10.17925/enr.2012.07.01.35

[8] Habib MA Paul B Alam MR Anwar N Karim MR Dhar PB. Non-motor symptoms of Parkinsons Disease patients attending at a tertiary care hospital of Bangladesh. Bangabandhu Sheikh Mujib Med Univ J. (2022) 14:121–4. doi: 10.3329/bsmmuj.v14i4.56610

[9] Huang L Wang Y Hong J. Traditional Chinese medicine for non-motor symptoms in Parkinson disease: a systematic review and meta-analysis of RCTs. Medicine. (2023) 102:e34425. doi: 10.1097/MD.0000000000034425, 37505124 PMC10378741

[10] Wang K Sun P Lv P Zhang P Li K Jiao B . Mind-body exercises for non-motor symptoms of patients with Parkinson’s disease: a systematic review and meta-analysis. Front Aging Neurosci. (2021) 13:314. doi: 10.3389/fnagi.2021.770920, 36226304 PMC9549381

[11] Bayer A Doumas M Stevenson R Carapellotti AM. The efficacy of dance for improving motor impairments, non-motor symptoms, and quality of life in Parkinson’s disease: a systematic review and meta-analysis (2020) 15:e0236820. doi: 10.1371/journal.pone.0236820, PMC7406058 32756578

[12] Vuletić V. Non-Motor Symptoms in Parkinson’s Disease Parkinson’s Disease. In: Demarin, V. (eds.) Mind and Brain. Springer, Cham. (2020). 109–18. doi: 10.1007/978-3-030-38606-1_9

[13] Li Q Zhu M Li Z Hao X Li M Wu C . Effect of acupuncture for non-motor symptoms in patients with Parkinson’s disease: a systematic review and meta-analysis. Front Aging Neurosci. (2022) 14:995850. doi: 10.3389/fnagi.2022.995850, 36275001 PMC9582755

[14] Zambetta ML Fernandes EB Kim A Russo TL Gianlorenço AC. Preclinical Parkinson’s disease models for non-motor symptoms: research recommendations from a systematic review. Life. (2025) 15:1034. doi: 10.3390/life15071034, 40724536 PMC12299557

[15] Xie D Zhou J Xu Y Shen Q. Non-motor symptoms are associated with REM sleep behavior disorder in Parkinson’s disease: a systematic review and meta-analysis. Neurol Sci. (2021) 42:47–60. doi: 10.1007/s10072-020-04769-9, 33025325

[16] Khan A Ezeugwa J Ezeugwu V. A systematic review of the associations between sedentary behavior, physical inactivity, and non-motor symptoms of Parkinson’s disease. PLoS One. (2023) 19:e0293382. doi: 10.1371/journal.pone.0293382 PMC10980241 38551932

[17] Li X Chen P Wang S Wang C Li B Liu H. Efficacy of acupuncture therapy in treating non-motor symptoms of Parkinson’s disease: a systematic review and network meta-analysis. Neuropsychiatr Dis Treat. (2025) 21:1799–822. doi: 10.2147/NDT.S541627 40910090 PMC12406037

[18] Maass A Reichmann H. Sleep and non-motor symptoms in Parkinson’s disease. J Neural Transm. (2013) 120:565–9. doi: 10.1007/s00702-013-0966-4, 23338671 PMC3611039

[19] Chen T Liu Y Deng Y Zhang S Teng S Cai B . "Effect of acupuncture on Parkinson’s disease". Translational Acupuncture Research. Cham: Springer International Publishing. (2019). 309–334.

[20] Dorsey ER Okun MS Bloem BR Sherer T. The emerging evidence of the Parkinson pandemic. J Parkinsons Dis. (2018) 8:S3–8. doi: 10.3233/JPD-181474, 30584159 PMC6311367

[21] Hsu WT Hsu CM Hung SC Hung SY. Acupuncture Improves sleep disorders and depression among patients with Parkinson's disease: a meta-analysis. Healthcare (Basel). (2023) 11:2042. doi: 10.3390/healthcare11142042 37510483 PMC10379076

[22] Yan M Fan J Liu X Li Y Wang Y Tan W . Acupuncture and sleep quality among patients with Parkinson disease: a randomized clinical trial. JAMA Netw Open. (2024) 7:e2417862. doi: 10.1001/jamanetworkopen.2024.17862, 38922617 PMC11208974

[23] Kluger BM Rakowski D Christian M Cedar D Wong B Crawford J . Randomized, controlled trial of acupuncture for fatigue in Parkinson’s disease. Mov Disord. (2016) 31:1027–32. doi: 10.1002/mds.26597, 27028133

[24] Fan JQ Lu WJ Tan WQ Liu X Wang YT Wang NB . Effectiveness of acupuncture for anxiety among patients with Parkinson disease: a randomized clinical trial. JAMA Netw Open. (2022) 5:e2232133. doi: 10.1001/jamanetworkopen.2022.32133, 36129711 PMC9494193

[25] Liu S Li H Zhang J Ma X Chen J Yang T . Acupuncture versus sham acupuncture in the treatment of insomnia for patients with Parkinson’s disease: a randomized controlled clinical trial. BMC Complement Med Ther. (2025) 25:278. doi: 10.1186/s12906-025-05009-3, 40684211 PMC12276649

[26] Pagonabarraga J Tinazzi M Caccia C Jost WH. The role of glutamatergic neurotransmission in the motor and non-motor symptoms in Parkinson’s disease: clinical cases and a review of the literature. J Clin Neurosci. (2021) 90:178–83. doi: 10.1016/j.jocn.2021.05.056, 34275546

